# Clinical implications of carotid artery intima media thickness assessment on cardiovascular risk stratification in hyperlipidemic Korean adults with diabetes: the ALTO study

**DOI:** 10.1186/s12872-015-0109-y

**Published:** 2015-10-06

**Authors:** Eun-Gyoung Hong, Jung Hun Ohn, Seong Jin Lee, Hyuk Sang Kwon, Sin Gon Kim, Dong Jun Kim, Dong Sun Kim

**Affiliations:** Division of Endocrinology and Metabolism, Department of Internal Medicine, Hallym University College of Medicine, Chuncheon, Korea; Division of Endocrinology and Metabolism, Department of Internal Medicine, Yeouido St. Mary’s Hospital, Seoul, Korea; Division of Endocrinology and Metabolism, Department of Internal Medicine, College of Medicine, Korea University, Seoul, Korea; Division of Endocrinology and Metabolism, Department of Internal Medicine, Inje University College of Medicine, Goyang, Korea; Division of Endocrinology and Metabolism, Department of Internal Medicine, Hanyang University Hospital, 222-1 Wangsimni-ro, Seoul, Seongdong-gu 133-792 Korea

**Keywords:** Subclinical atherosclerosis, Diabetes, Hyperlipidemia, Carotid artery ultrasonography

## Abstract

**Background:**

The primary objective was to investigate prevalence of subclinical atherosclerosis in Korean individuals with diabetes and hyperlipidemia. Association of subclinical atherosclerosis with cardiovascular risk was assessed.

**Methods:**

Assessments of carotid artery intima media thickness (cIMT) and atheromatous plaque were done using B-mode ultrasonography. Subclinical atherosclerosis was diagnosed based on presence of plaque, and/or increased cIMT versus mean cIMT reference values for Korean healthy controls. Atherosclerosis risk factors were analyzed using United Kingdom Prospective Diabetes Study (UKPDS) risk engine and Framingham Risk Score.

**Results:**

In total, 355 patients were included; increased mean cIMT was observed in 15.3 % of patients, 69 % had >1 carotid artery plaque, and 72.7 % were diagnosed with subclinical atherosclerosis. In total, 60 % of subjects were taking statins, with low-density lipoprotein cholesterol level maintained ~80 mg/dL at enrollment. Carotid artery measures were well correlated with UKPDS and Framingham risk scores. Prevalence of subclinical atherosclerosis in the low risk group (<15 % 10-year UKPDS-predicted coronary heart disease risk) was 64.7 %; higher than predicted in previous studies. In multivariate analysis, advanced age was a significant risk factor for subclinical atherosclerosis in men and women, while increased waist circumference and longer diabetes duration were independent predictors only in women.

**Conclusion:**

Subclinical atherosclerosis is more prevalent among individuals with both diabetes and hyperlipidemia than in diabetic patients without additional cardiovascular risk factors. As conventional risk engines, based on modifiable risk factors may underestimate cardiovascular risk, early non-invasive carotid artery imaging screening may be warranted for patients with diabetes and hyperlipidemia, especially if they are elderly, have central obesity or have long duration of diabetes.

**Trial registration:**

www.clinicaltrials.gov NCT01264263

**Electronic supplementary material:**

The online version of this article (doi:10.1186/s12872-015-0109-y) contains supplementary material, which is available to authorized users.

## Background

Patients with diabetes show increased morbidity and mortality from atherosclerotic diseases [1]. Coronary artery disease in diabetic patients is associated with traditional risk factors, including dyslipidemia, hypertension, poor glycemic control, obesity, and smoking [2]. Furthermore, atherosclerotic complications in diabetes are mediated by abnormalities in endothelial and vascular smooth muscle cell function caused by metabolic abnormalities such as hyperglycemia, increase of free fatty acids, and insulin resistance [3–5].

Sudden death is a common manifestation of coronary heart disease (CHD) [6]. Therefore, there is a great interest in identifying asymptomatic individuals at risk who would be candidates for more intensive and evidence-based medical interventions [7]. B-mode ultrasound measurement of carotid intima media thickness (cIMT) is a non-invasive method for estimating atherosclerotic burden, with increased cIMT or the presence of atheromatous plaque predicting CHD and stroke after adjustment for other risk factors [8, 9]. In a study of a multiethnic population, 23 % of individuals classified as being at low risk for CHD by Framingham Risk Score had subclinical atherosclerosis, emphasizing the need for non-invasive assessment of atherosclerotic burden in risk prediction [10].

Consistent with the increased incidence of cardiovascular disease (CVD) in diabetic patients, cIMT is increased in patients with diabetes compared with those without diabetes. In a study of 826 subjects aged 40–79 years, impaired glucose tolerance and diabetes were independent predictors of advanced stenotic carotid atherosclerosis [11]. Also, cIMT was significantly greater in elderly patients (aged 67 years on average) with diabetes than in control subjects [12]. In the Atherosclerosis Risk in Communities (ARIC) study, patients with diabetes had significantly increased cIMT compared with those without diabetes regardless of prior myocardial infarction (MI), and non-diabetic patients with MI had cIMT similar to that of diabetic patients without MI [13]. In a study of Koreans, 43.5 % of patients with diabetes had increased cIMT, and it was associated with elevated risk of CHD, stroke, and peripheral artery disease [14]. However, there was no further evaluation of risk stratification or plaque status.

Therefore, in contrast to previous studies, we recruited Korean patients who simultaneously had two major cardiovascular (CV) risk factors, diabetes and hyperlipidemia, but did not have symptomatic CVD or any other major vascular disease. We evaluated the prevalence of subclinical atherosclerosis in this specific patient group with high CV risk. Subclinical atherosclerosis was defined by the increase of cIMT or the presence of atheromatous plaque, according to the result of carotid artery ultrasonography. We also investigated the associated risk factors for subclinical atherosclerosis and analyzed risk stratification by conventional CV risk predictors.

## Methods

The Prevalence of Subclinical Atherosclerosis and its Associated Factors in Hyperlipidemic Korean Adults with Diabetes (ALTO) study (NCT01264263) is a multicenter, cross-sectional investigation of subclinical atherosclerosis by cIMT and its associated factors in six university-affiliated hospitals in Korea.

### Study population

All study participants were randomly enrolled from six university-affiliated hospitals on an endocrinologic basis. The following ethics committees/institutional review boards (IRBs) of the participating institutions approved the study protocol: Hallym University Dongtan Sacred Heart Hospital Ethics Committee/IRB, Hwaseong-si, Gyeonggi-do, Korea; Hallym University Sacred Heart Hospital Ethics Committee/IRB, Anyang-si, Gyeonggi-do, Korea; The Catholic University of Korea, Yeouido St. Mary’s Hospital Ethics Committee/IRB, Seoul, Korea; Korea University Anam Hospital Ethics Committee/IRB, Seoul, Korea; Inje University Ilsan Paik Hospital Ethics Committee/IRB, Goyang-si, Gyeonggi-do, Korea; Hanyang University Hospital Ethics Committee/IRB, Seoul, Korea. All participants provided written informed consent.

Patients aged 20–80 years who had diabetes and hyperlipidemia were enrolled. We excluded all participants with a prior history of any CVD defined as MI, silent MI, angina, prior percutaneous coronary intervention or coronary artery bypass graft surgery, or history of stroke or peripheral artery disease. Participants who had malignancy, chronic inflammatory disease, impaired renal or liver function, or history of taking non-steroidal anti-inflammatory drugs that could affect high-sensitivity C-reactive protein (hsCRP) level, and patients who used steroids for 7 consecutive days within the previous 90 days were also excluded. Diabetes was defined as the use of any anti-diabetic drugs, including oral hypoglycemic agents and insulin, or fasting plasma glucose concentration ≥126 mg/dL, or plasma glucose ≥200 mg/dl on a 2-hour oral glucose tolerance test. Hyperlipidemia was defined as total cholesterol ≥200 mg/dL, triglycerides ≥150 mg/dL, or low-density lipoprotein cholesterol (LDL-C) ≥100 mg/dL according to the National Cholesterol Education Program (NCEP) Adult Treatment Panel III guidelines, or if the patient was taking anti-hyperlipidemic medications.

### Assessment of risk factors

Demographics, lifestyle, and medical history were obtained using a self-reported questionnaire. Body weight and height were measured with subjects wearing light-weight clothes. Body mass index (BMI) was calculated as weight (kg) divided by height (m). Waist circumference was measured by a plastic tape measure placed in the middle between the lowest margin of the ribs and the upper margin of the iliac crest with subjects standing with their feet 30 cm apart. Blood pressure was measured twice on the left arm after the subjects rested for at least 10 min in a sitting position, and the mean value of the two measurements was used. Smoking status was evaluated by the US Centers for Disease Control and Prevention, based on questionnaire responses regarding smoking habit. Subjects who had smoked at least 100 cigarettes in their lifetime and who, at the time of survey, smoked either every day or some days were defined as a “current smoker”. Subjects who reported smoking at least 100 cigarettes in their lifetime but who did not smoke at all at the time of the survey were defined as a “former smoker”. Patients who had never smoked a cigarette or who smoked fewer than 100 cigarettes were defined as “never a smoker”.

All of the blood samples were taken after 12–16 hours of fasting and analyzed within 24 hours of transportation to the central laboratory (Green Cross Reference Laboratory, Seoul, Korea). Fasting plasma glucose level was measured by hexokinase method (Modular Analytics, Roche Diagnostics, Mannheim, Germany). High-performance liquid chromatography (Variant II TURBO, Bio Rad, Hercules, California, USA) was used for the measurement of glycated hemoglobin (HbA1c) level and total cholesterol, triglyceride, high-density lipoprotein cholesterol (HDL-C) and LDL-C were measured by enzymatic colorimetric assay (Modular Analytics, Roche Diagnostics, Mannheim, Germany). Apolipoprotein A1, apolipoprotein B, hsCRP, urinary albumin and creatinine for the diagnosis of albuminuria were measured by immunoturbidimetric assay (Modular Analytics, Roche Diagnostics, Mannheim, Germany).

### Carotid ultrasonography

Carotid artery atherosclerosis was determined by one examiner for all patients across all six sites, using high-resolution B-mode ultrasound (M-Turbo^®^, SonoSite, Washington, Bothell, USA), according to protocol [15]. A transverse scan was followed by a longitudinal circumferential scan at 12 well-defined segments, the near and the far walls of the right and left common carotid, bulb, and internal carotid arteries. Circumferential plaque was screened from three imaging angles: anterior, lateral, and posterior. cIMT was measured at the far walls of the distal 1 cm of each common carotid artery at three imaging angles, and the mean and maximum cIMT values were measured by leading edge-to-edge method. Plaque was defined as a focal structure encroaching into the arterial lumen of at least 0.5 mm or 50 % of the surrounding cIMT value or a demonstrating thickness >1.5 mm as measured from the media-adventitia interface to the intima-lumen interface [16].

The mean maximum value of cIMT in each study subject (described as mean cIMT) was calculated based on the average of maximum cIMT values within the 12 arterial wall segments. The mean value was then compared with the mean value (average value of maximal cIMT) in 757 healthy Korean subjects, with corresponding age and sex as the reference [17]; these cut-off values were used to define subclinical atherosclerosis in the current study (Additional file 1: Table S1).

### United Kingdom prospective diabetes study and Framingham Risk Score calculation

The United Kingdom Prospective Diabetes Study (UKPDS) risk engine (ver. 2.0) was downloaded from the website (http://ukpds-risk-engine.software.informer.com/2.0/) and used to analyze the data. The Framingham Risk Score was calculated using the equation for estimating 10-year CVD risk [18].

### Statistical analysis

Statistical analyses were performed using SPSS V16.0. For continuous variables, an analysis of covariance was used, and the chi-squared test was used in the categorical data analysis. Continuous variables which did not show normal distribution were log-transformed before statistical tests were performed. All statistical tests were two-sided. Pearson correlation analysis was used to determine the relationships between CV risk scores and cIMT measures. The association between conventional risk scores and subclinical atherosclerosis was evaluated through calculating the area under the receiver operating characteristic (ROC) curve at different levels of risk scores. The statistical significance of the difference in area under the curve (AUC) was calculated by the MedCalc software (http://www.medcalc.org/). Predictors of subclinical atherosclerosis were obtained by logistic regression analysis, following consideration of additive or confounding factors. Values in the text are the mean ± standard deviation, unless otherwise specified.

## Results

### Characteristics of study subjects

A total of 362 diabetic patients aged 20–80 years with hyperlipidemia were initially enrolled. The data of 355 participants without established or previous CVD were eligible for analysis after exclusion of seven patients who did not match the protocol guidelines. Clinical and laboratory characteristics of study subjects were analyzed by sex (Table 1). Women (*n* = 178, 60.2 ± 10.2 years) were significantly older than men (*n* = 177, 55.9 ± 10.9 years) but the mean durations of diabetes were not significantly different (women 8.1 ± 7.3 vs men 7.5 ± 7.3 years, *p* = 0.143). Also, mean BMI was similar, but waist circumference was significantly bigger in men (90.6 ± 7.0 vs women 86.3 ± 10.0 cm). Fasting plasma glucose level and HbA1c of total study subjects were 136.2 ± 40.7 mg/dL and 7.4 ± 1.1 %, respectively, with no significant differences between men and women. In the lipid analysis, both total cholesterol and HDL-C were significantly higher in women than in men (169.8 ± 34.3 vs 160.9 ± 32.9 mg/dL, 54.8 ± 12.8 vs 49.3 ± 11.2 mg/dL, respectively). hsCRP was 0.5 ± 0.9 mg/L and mean random urine albumin to creatinine ratio was not significant. Among men, 140 (78.7 %) of 177 were current or previous smokers, but among women only eight (4.5 %) of 178 were current or previous smokers. Statins were prescribed in 237 (66.8 %) of 355 study subjects.

**Table 1:** Baseline clinical and laboratory characteristics of study participants

	Men	Women	p-value
	(*n* = 178)	(*n* = 177)	
Age (years)	55.9 ± 10.9	60.2 ± 10.2	<0.001
Body mass index (kg/m^2^)	25.5 ± 3.1	25.2 ± 4.0	0.468
Waist circumference (cm)	90.6 ± 7.0	86.3 ± 10.0	<0.001
Duration of diabetes (years)	7.5 ± 7.3	8.6 ± 7.3	0.143
Systolic blood pressure (mmHg)	128.7 ± 13.5	128.2 ± 14.2	0.726
Diastolic blood pressure (mmHg)	79.3 ± 9.6	78.2 ± 8.9	0.257
Smoking, (never/ex/current)	38/72/68	169/2/6	<0.001
Family history of CHD (%)	12 (6.7)	9 (5.1)	0.654
Menopause	–	139 (78.5)	–
Atrial fibrillation (%)	2 (1.1)	2 (1.1)	0.686
ACE inhibition (%)	61 (34.3)	70 (39.5)	0.323
Antiplatelet medication (%)	51 (28.7)	44 (24.9)	0.472
Statin use (%)	114 (64)	123 (69.5)	0.311
Total cholesterol (mg/dL)	160.9 ± 32.9	169.8 ± 34.3	0.013
Triglycerides (md/dL)	155.3 ± 116.9	138.1 ± 81.3	0.108
HDL-C (mg/dL)	49.3 ± 11.2	54.8 ± 12.8	<0.001
LDL-C (mg/dL)	85.7 ± 28.7	91.3 ± 28.0	0.061
ApoB (mg/dL)	71.3 ± 21.4	71.8 ± 20.3	0.813
ApoA1 (mg/dL)	130.0 ± 27.4	137.8 ± 2.8	0.006
Fasting blood glucose (mg/dL)	137.0 ± 41.9	135.3 ± 39.6	0.694
HbA1c (%)	7.4 ± 1.1	7.5 ± 1.2	0.449
hsCRP (mg/L)^a^	0.5 ± 0.9	0.5 ± 0.9	0.618
Albumin/creatinine ratio^a^	12.1 ± 26.7	12.1 ± 28.6	0.239^b^

Values are mean ± SD; ^a^Median ± IQR; ^b^The variable was log transformed before statistical analysis

ACE, angiostensin-converting enzyme; Apo, apolipoprotein; CHD, coronary heart disease; HbA1c, glycated hemoglobin; HDL-C, high density lipoprotein cholesterol; hsCRP, high-sensitivity C-reactive protein; IQR, interquartile range; LDL-C, low density lipoprotein cholesterol; SD, standard deviation

### Prevalence of subclinical atherosclerosis in patients with type 2 diabetes and dyslipidemia

**Fig. 1:**
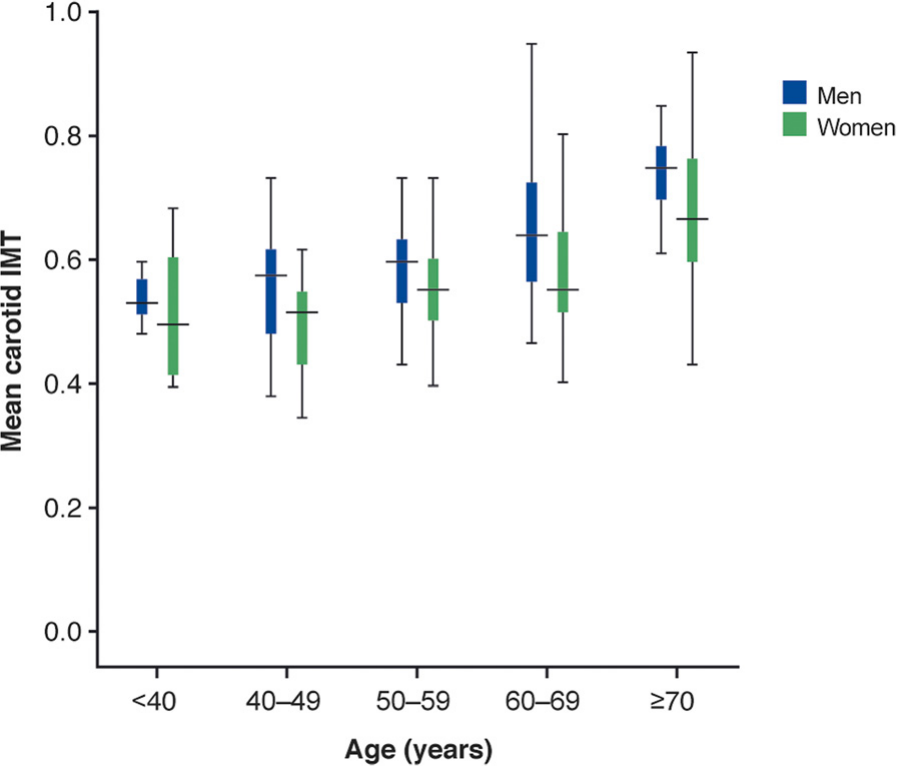
Distribution of mean cIMT according to age and sex category

The mean and maximum cIMT and the number of plaques in study subjects are shown in Table 2 and the mean cIMT according to age and sex is shown in Fig. 1. The mean cIMT was 0.61 ± 0.11 mm in men and 0.59 ± 0.12 mm in women (*p* = NS). A total of 54 (15.3 %) of 354 study subjects had increased cIMT when compared with the healthy control reference values [17]. The mean number of plaques was 1.89 ± 2.46 in men and 1.42 ± 1.59 in women (*p* = 0.034) (Table 2). Sixty-nine percent of all patients were found to have one or more plaques (70.8 % in men and 67.2 % in women) (Table 2).

**Table 2:** cIMT and plaque measures in patients with diabetes and hyperlipidemia

	Total	Men		Women	p-value	
Age (years)		n		n		
Mean cIMT (mm)						
<40	0.522 ± 0.086	12	0.525 ± 0.069	7	0.514 ± 0.114	
40–49	0.532 ± 0.090	39	0.555 ± 0.089	21	0.489 ± 0.076	
50–59	0.586 ± 0.101	60	0.589 ± 0.084	54	0.582 ± 0.118	
60–69	0.612 ± 0.114	44	0.650 ± 0.124	61	0.585 ± 0.098	
≥70	0.691 ± 0.116	23	0.737 ± 0.066	33	0.677 ± 0.122^†^	
Total	0.598 ± 0.116	178	0.610 ± 0.110	177	0.587 ± 0.119	0.082
Max cIMT (mm)						
<40	0.526 ± 0.087	12	0.532 ± 0.072	7	0.514 ± 0.114	
40–49	0.532 ± 0.090	39	0.555 ± 0.090	21	0.489 ± 0.076	
50–59	0.600 ± 0.132	60	0.603 ± 0.120	54	0.597 ± 0.145	
60–69	0.617 ± 0.125	44	0.661 ± 0.145	61	0.585 ± 0.098	
≥70	0.705 ± 0.141	23	0.736 ± 0.157	34	0.684 ± 0.126^a^	
Total	0.606 ± 0.134	178	0.619 ± 0.137	177	0.593 ± 0.130	0.062
Plaque (presence, %)						
<40	8/19 (42.1)	12	6/12 (50.0)	7	2/7 (28.6)	
40–49	30/60 (50.0)	39	20/39 (51.3)	21	10/21 (47.6)	
50–59	74/114 (64.9)	60	41/60 (68.3)	54	33/54 (61.1)	
60–69	84/105 (80.0)	44	39/44 (88.6)	61	45/61 (73.8)	
≥70	49/57 (86.0)	23	20/23 (87.0)	34	29/34 (85.3)	
Total	245/355 (69.0)	178	126/178 (70.8)	177	119/177 (67.2)	0.469
Plaque (number)						
<40	0.526 ± 0.697	12	0.583 ± 0.669	7	0.429 ± 0.787	
40–49	0.700 ± 0.830	39	0.718 ± 0.826	21	0.667 ± 0.856	
50–59	1.377 ± 1.502	60	1.600 ± 1.639	54	1.130 ± 1.304	
60–69	2.257 ± 2.756	44	3.273 ± 3.681	61	1.525 ± 1.468	
≥70	2.509 ± 2.213	23	2.696 ± 2.363	34	2.382 ± 2.132	
Total	1.659 ± 2.083	178	1.893 ± 2.462	177	1.424 ± 1.587	0.034

^a^One study subject who had mean and maximum cIMT of 2 mm was excluded as an outlier

cIMT, carotid intima media thickness

Based on comparison versus healthy Korean subjects with corresponding age and sex [17], the prevalence of subclinical atherosclerosis was 72.7 % in total participants (74.7 % in men vs 70.6 % in women, respectively) (Table 3).

**Table 3:** Prevalence of subclinical atherosclerosis^a^ by age and sex difference

Age (years)	Total (%)	Men (%)	Women (%)
<40	12/19 (63.2)	8/12 (66.7)	4/7 (57.1)
40–49	34/60 (56.7)	24/39 (61.5)	10/21 (47.6)
50–59	79/114 (69.3)	42/60 (70.0)	37/54 (68.5)
60–69	84/105 (80.0)	39/44 (88.6)	45/61 (73.8)
≥70	49/57 (86.0)	20/23 (87.0)	29/34 (85.3)
Total	258/355 (72.7)	133/178 (74.7)	125/177 (70.6)

^a^Subclinical atherosclerosis is defined as more than the average age- and sex-matched mean cIMT or the presence of carotid plaque

cIMT, carotid intima media thickness

### Correlation analysis between cIMT measures and conventional CV risk engine score

The correlations between the two most popular CV risk assessment engines, UKPDS and Framingham risk scoring systems, and cIMT measures, were evaluated. Mean and maximum cIMT and the number of carotid plaques were significantly correlated with both UKPDS and Framingham Risk Scores (Additional file 1: Table S2). ROC curve analysis showed a slight trend towards better correlation between UKPDS and cIMT measures from AUC calculation, compared with Framingham risk prediction, although it was not statistically significant (AUC 0.667 vs 0.636, respectively; *p* = 0.189, Additional file 1: Figure S1).

We separated subjects into low- and high-risk groups by the cut-off point of 15 % 10-year CHD risk according to the UKPDS risk engine, and compared cIMT and plaque status in each study group. As shown in Additional file 1: Table S3, mean cIMT and the presence and the number of plaques were all significantly higher in the high-risk group.

### The prevalence of subclinical atherosclerosis by UKPDS 10-year CHD risk stratification

Table 4 shows the prevalence of subclinical atherosclerosis according to UKPDS risk group by sex. Approximately 81.5 % of participants estimated to be at high risk by the UKPDS risk engine had subclinical atherosclerosis. However, 64.7 % of the low risk patients also had subclinical atherosclerosis.

**Table 4:** Prevalence of subclinical atherosclerosis according to UKPDS risk group

	Men (%)			Women (%)		
Age (years)	Low risk	High risk	p-value	Low risk	High risk	p-value
<40	7/11 (63.6)	1/1 (100.0)		4/7 (57.1)	-	
40–49	20/30 (66.7)	4/9 (44.4)		9/19 (47.4)	1/2 (50.0)	
50–59	18/28 (64.3)	24/32 (75.0)		30/46 (65.2)	7/8 (87.5)	
60–69	3/3 (100.0)	36/41 (87.8)		25/35 (71.4)	20/26 (76.9)	
≥70	0	20/23 (87.0)		1/3 (33.3)	28/31 (90.3)	
Subtotal	48/72 (66.7)	85/106 (80.2)	0.042	69/110 (62.7)	56/67 (83.6)	0.003

### Predictors of subclinical atherosclerosis in patients with type 2 diabetes and hyperlipidemia

We stratified study subjects by sex and explored clinical predictors of subclinical atherosclerosis in univariate and multivariate logistic regression models (Additional file 1: Tables S4 and S5). In men, only advanced age was a significant predictor of subclinical atherosclerosis (odds ratio [OR] 1.05, 95 % confidence interval [CI] 1.017–1.084, *p* < 0.005), while advanced age (OR 1.061, 95 % CI 1.025–1.098, *p* = 0.001), longer duration of diabetes (OR 1.098, 95 % CI 1.02–1.181, *p* = 0.013) and increased waist circumference (OR 1.051, 95 % CI 1.008–1.096, *p* = 0.02) were independent predictors of subclinical atherosclerosis in women.

## Discussion

A total of 355 diabetic patients with hyperlipidemia were recruited in our study and underwent B-mode ultrasound measurement of cIMT. The main finding was the high prevalence of subclinical atherosclerosis in Korean patients with diabetes and hyperlipidemia; 72.7 % of study patients had asymptomatic atherosclerosis with an increase in cIMT and/or carotid artery plaques assessed by non-invasive imaging.

Patients with diabetes and hyperlipidemia are classified as high-risk groups by the NCEP guideline, and the committee recommends lower LDL-C level treatment goals regardless of atheroma burden [19]. Evaluation of atherosclerotic burden by non-invasive imaging has not been incorporated in clinical practice. Also, current guidelines for primary prevention of CVD recommend the use of traditional CV risk factors in initial risk stratification [19–21]. However, the most commonly used Framingham Risk Score is reported to underestimate CV risk in patients with diabetes [22] and the UKPDS risk engine may not provide reliable risk estimate among patients with diabetes in the era of widespread use of statins that modify cholesterol levels [23]. Of note, several large-scale studies have addressed the relationship between cIMT and CV events. In the ARIC study, cIMT ≥1 mm was associated with an increase in CHD by five-fold in women and two-fold in men after adjusting for age, social group, and race [24]. The Cardiovascular Health Study reported the elevated risk of MI or stroke with increase in cIMT among study participants >65 years of age [25]. In the Rotterdam Study, carotid plaques, cIMT, aortic atherosclerosis, and lower-extremity atherosclerosis were equally predictive of future MI [26, 27]. Consequently, a recent guideline (Screening for Heart Attack Prevention and Education) recommends the non-invasive screening of all asymptomatic men 45–75 years of age and asymptomatic women 55–75 years of age at initial risk stratification [28].

In our study, 60 % of study subjects were taking statins and LDL-C was already maintained around 80 mg/dL at enrollment. In the low-risk group, estimated to have <15 % 10-year CHD risk by the UKPDS risk engine, a higher proportion of patients (64.7 %) had subclinical atherosclerosis. This suggests that the conventional risk-prediction algorithm based on modifiable risk factors may underestimate CV risk in patients with diabetes and hyperlipidemia, especially when LDL-C is maintained at the currently recommended target. This may also emphasize the value of non-invasive arterial imaging in predicting patient CV risk, and the need for further research to override the limits of the current approach based on traditional risk factors.

Additional interesting findings were shown in our study. The mean cIMT in this study population was less than the value in healthy Koreans, who were also assessed using ultrasound high-resolution B-mode carotid artery imaging [17], with the prevalence of subclinical atherosclerosis based on cIMT only (more than average of age- and sex-specific normal values in Koreans) found to be 15.3 % in the current study. Medications such as statins, anti-hypertensives, oral anti-diabetic drugs, and anti-platelet agents are known to have a variety of cardioprotective effects, including, in some cases, positive effects on atherosclerosis [29, 30], and patients were not excluded from this study based on their use of such medications. For example, the biguanide glucose-lowering agent, metformin, has been shown to have a number of cardioprotective effects, including reduction in overall CV mortality [30]; however, the direct effect of metformin on cIMT is weak [31], and metformin use is therefore considered unlikely to have had a significant impact on results in the present study. Conversely, use of statins has been shown to decrease progression rate, or even regress, cIMT [32]. Therefore, the findings that 60 % of our study subjects were taking statins and that the mean cIMT among statin users was smaller than that in non-users (although not statistically significant, Additional file 1: Table S6), may partly explain the smaller cIMT in our study populations. This result demonstrated that the strict control of LDL-C could differently affect cIMT and established plaques [31, 32]. Therefore carotid artery plaque presence seems to work as a robust measure of carotid artery atherosclerosis in populations on various medications affecting cIMT, because although plaques may regress after treatment with medication, complete resolution will be rare, especially in patients with a high risk of CVD.

Furthermore, aging was the most significant predictor of subclinical atherosclerosis, and those who were centrally obese and had diabetes for long periods, especially among women, were more likely to have subclinical atherosclerotic disease. Additional biochemical or clinical variables were not predictive of subclinical atherosclerosis, which may be attributable to the relatively homogeneous population characteristics, i.e. patients already had two strong risk factors for atherosclerosis: diabetes and hyperlipidemia.

We surveyed the prevalence of subclinical atherosclerosis in Korean patients with diabetes and hyperlipidemia who were at high risk for future CV events. Carotid artery imaging with B-mode ultrasound is a validated method for identifying subclinical atherosclerosis, and this technique was applied by one skilled sonographer for all patients across all six sites, to minimize inter- and intra-site technical variability. A limitation of our study is that a control group, without diabetes or hyperlipidemia, was not included in the survey. Furthermore, patients were included irrespective of whether they had other CV risk factors, such as overweight/obesity and hypertension. Mean BMI values were found to be slightly higher than the upper-normal bracket of 25 kg/m^2^ in both men and women in this study (Table 1); this is perhaps unsurprising given the known association between overweight and type 2 diabetes.

Conventional risk engines are based on clinical risk factors and biochemical parameters that can be modified by medications, and may underestimate CV risk in high-risk patients. Based on the high prevalence of subclinical atherosclerosis in diabetic patients with hyperlipidemia, we can conclude that early screening for the detection of subclinical atherosclerosis and more aggressive management are warranted for patients with diabetes and hyperlipidemia, especially if they are elderly, have central obesity and have a long duration of diabetes. Carotid artery plaque presence may be a more reliable measure of atherosclerotic burden than cIMT, as medications such as statins may differentially affect the plaque and the thickness of the carotid arterial wall.

## Additional file

Additional file 1: Table S1. Mean cIMT values that were used as cutoff values for determining subclinical atherosclerosis. **Table S2**. Correlation between 10-year cardiovascular risk and cIMT measures UKPDS Framingham Risk Score. **Table S3**. cIMT measures between UKPDS coronary heart disease risk groups by gender and age. **Table S4**. Univariate predictors of subclinical atherosclerosis. **Table S5**. Multivariate independent predictors of subclinical atherosclerosis. **Table S6**. Mean cIMT according to age and the status of statin use. **Figure S1**. Receiver operating characteristic curve analysis of conventional risk scores in predicting subclinical atherosclerosis (*p* = 0.189). (DOCX 787 kb)

## Abbreviations

AUC: Area under the curve
BMI: Body mass index
CHD: Coronary heart disease
CI: Confidence interval
cIMT: carotid intima media thickness
CV: Cardiovascular
CVD: Cardiovascular disease
HbA1C: Glycated hemoglobin
HDL-C: High-density lipoprotein cholesterol
hsCRP: High-sensitivity C-reactive protein
LDL-C: Low-density lipoprotein cholesterol
MI: Myocardial infarction
NCEP: National Cholesterol Education Program
OR: Odds ratio
ROC: Receiver operating characteristic
UKPDS: United Kingdom Prospective Diabetes Study
