# Supplementary material for: Clinical implications of carotid artery intima media thickness assessment on cardiovascular risk stratification in hyperlipidemic Korean adults with diabetes: the ALTO study
Source: BMC Cardiovasc Disord. 2015 Oct 6;15:114. doi: 10.1186/s12872-015-0109-y (PMC4595332; doi:10.1186/s12872-015-0109-y)
Supplement: Additional file 1: Table S1. — Mean cIMT values that were used as cutoff values for determining subclinical atherosclerosis. Table S2. Correlation between 10-year cardiovascular risk and cIMT measures UKPDS Framingham Risk Score. Table S3. cIMT measures between UKPDS coronary heart disease risk groups by gender and age. Table S4. Univariate predictors of subclinical atherosclerosis. Table S5. Multivariate independent predictors of subclinical atherosclerosis. Table S6. Mean cIMT according to age and the status of statin use. Figure S1. Receiver operating characteristic curve analysis of conventional risk scores in predicting subclinical atherosclerosis (p = 0.189). (DOCX 787 kb) [file 12872_2015_109_MOESM1_ESM.docx]

Clinical implications of carotid artery intima media thickness assessment on cardiovascular risk stratification in hyperlipidemic Korean adults with diabetes: the ALTO study

Eun-Gyoung Hong, Jung Hun Ohn, Seong Jin Lee, Hyuk Sang Kwon, Sin Gon Kim, Dong Jun Kim and Dong Sun Kim

Table S1. Mean cIMT values that were used as cutoff values for determining subclinical atherosclerosis

| **Age (years)** | **Men** | **Women** |
| --- | --- | --- |
| <40 | 0.57 | 0.57 |
| 40–49 | 0.61 | 0.60 |
| 50–59 | 0.72 | 0.67 |
| 60–69 | 0.77 | 0.71 |
| ≥70 | 0.88 | 0.76 |

Modified from Cho YL, Kim DJ, Kim HD, et al. Reference values of carotid artery intima-media thickness and association with atherosclerotic risk factors in healthy subjects in Korea. Korean J Med. 2003;64:275-83.

cIMT, carotid intima media thickness

Table S2. Correlation between 10-year cardiovascular risk and cIMT measures UKPDS Framingham Risk Score

|  | **CHD** | | **Fatal CHD** | | **Stroke** | | **Fatal stroke** | |
| --- | --- | --- | --- | --- | --- | --- | --- | --- |
|  | **r** | **p** | **r** | **p** | **r** | **p** | **r** | **p** |
| Men |  |  |  |  |  |  |  |  |
| Mean cIMT (mm) | 0.372 | <0.001 | 0.359 | <0.001 | 0.348 | <0.001 | 0.341 | <0.001 |
| Max cIMT (mm) | 0.361 | <0.001 | 0.347 | <0.001 | 0.341 | <0.001 | 0.325 | <0.001 |
| Number of plaques | 0.461 | <0.001 | 0.459 | <0.001 | 0.431 | <0.001 | 0.470 | <0.001 |
| Women | | | | | | | | |
| Mean cIMT (mm) | 0.350 | <0.001 | 0.338 | <0.001 | 0.332 | <0.001 | 0.314 | <0.001 |
| Max cIMT (mm) | 0.327 | <0.001 | 0.315 | <0.001 | 0.307 | <0.001 | 0.290 | <0.001 |
| Number of plaques | 0.319 | <0.001 | 0.318 | <0.001 | 0.398 | <0.001 | 0.363 | <0.001 |

|  | **CHD** | | **MI** | | **Stroke** | | **CVD** | |
| --- | --- | --- | --- | --- | --- | --- | --- | --- |
|  | **r** | **p** | **r** | **p** | **r** | **p** | **r** | **p** |
| Men |  |  |  |  |  |  |  |  |
| Mean cIMT (mm) | 0.336 | <0.001 | 0.244 | 0.001 | 0.361 | <0.001 | 0.362 | <0.001 |
| Max cIMT (mm) | 0.330 | <0.001 | 0.263 | <0.001 | 0.343 | <0.001 | 0.357 | <0.001 |
| Number of plaques | 0.436 | <0.001 | 0.419 | <0.001 | 0.466 | <0.001 | 0.445 | <0.001 |
| Women | | | | | | | | |
| Mean cIMT (mm) | 0.272 | <0.001 | 0.242 | 0.001 | 0.321 | <0.001 | 0.372 | <0.001 |
| Max cIMT (mm) | 0.255 | 0.001 | 0.223 | 0.003 | 0.303 | <0.001 | 0.350 | <0.001 |
| Number of plaques | 0.125 | 0.097 | 0.111 | 0.140 | 0.310 | <0.001 | 0.273 | <0.001 |

CHD, coronary heart disease; cIMT, carotid intima media thickness; CVD, cardiovascular disease; MI, myocardial infarction; UKPDS, United Kingdom Prospective Diabetes Study.

Table S3. cIMT measures between UKPDS coronary heart disease risk groups by gender and age

|  | **Men** | | | | | **Women** | | | | | |
| --- | --- | --- | --- | --- | --- | --- | --- | --- | --- | --- | --- |
|  | **Low risk** | | **High risk** | | **p-value** | **Low risk** | | | **High risk** | | **p-value** |
| **Age (years)** | **n** |  | **n** |  |  | **n** | |  | **n** |  |  |
| Mean cIMT (mm) |  |  |  |  |  |  | |  |  |  |  |
| <40 | 11 | 0.515 ± 0.061 | 1 | 0.645 |  | 7 | | 0.514 ± 0.114 | – |  |  |
| 40–49 | 30 | 0.557 ± 0.098 | 9 | 0.549 ± 0.058 |  | 19 | | 0.480 ± 0.072 | 2 | 0.583 ± 0.046 |  |
| 50–59 | 28 | 0.576 ± 0.079 | 32 | 0.600 ± 0.088 |  | 46 | | 0.563 ± 0.098 | 8 | 0.690 ± 0.164 |  |
| 60–69 | 3 | 0.642 ± 0.238 | 41 | 0.650 ± 0.117 |  | 35 | | 0.592 ± 0.106 | 26 | 0.575 ± 0.087 |  |
| ≥70 | 0 | – | 23 | 0.712 ± 0.106 |  | 3 | | 0.623 ± 0.159 | 30 | 0.682 ± 0.120^†^ |  |
| Subtotal | 72 | 0.561 ± 0.095 | 106 | 0.640 ± 0.112 | <0.001 | 110 | | 0.556 ± 0.106 | 66 | 0.638 ± 0.123 | <0.001 |
| Total | 0.608 ± 0.112 | | | |  | 0.597 ± 0.182 | | | | |  |
| Presence of plaque (%) |  | |  | |  | |  | |  | |  |
| <40 | 6/11 (54.5) | | 0/1 (0.0) | |  | 2/7 (28.6) | | | – | |  |
| 40–49 | 16/30 (53.3) | | 4/9 (44.4) | |  | 9/19 (47.4) | | | ½ (50.0) | |  |
| 50–59 | 18/28 (64.3) | | 23/32 (71.9) | |  | 28/46 (60.9) | | | 5/8 (62.5) | |  |
| 60–69 | 3/3 (100.0) | | 36/41 (87.8) | |  | 25/35(71.4) | | | 20/26 (76.9) | |  |
| ≥70 | 0 | | 20/23 (87.0) | |  | 1/3(33.3) | | | 28/31 (90.3) | |  |
| Subtotal | 43/72 (59.7) | | 83/106 (78.3) | | 0.011 | 65/110(59.1) | | | 54/67 (80.6) | | 0.003 |
| Total |  | |  | |  |  | | |  | |  |
| Plaque (number) |  | |  | |  |  | | |  | |  |
| <40 | 11 | 0.636 ± 0.674 | 1 | 0 |  | 7 | | 0.429 ± 0.787 | – |  |  |
| 40–49 | 30 | 0.700 ± 0.794 | 9 | 0.779 ± 0.972 |  | 19 | | 0.684 ± 0.885 | 2 | 0.500 ± 0.707 |  |
| 50–59 | 28 | 1.464 ± 1.551 | 32 | 1.719 ± 1.727 |  | 46 | | 1.109 ± 1.320 | 8 | 1.250 ± 1.282 |  |
| 60–69 | 3 | 2.333 ± 1.155 | 41 | 3.342 ± 3.799 |  | 35 | | 1.314 ± 1.157 | 26 | 1.808 ± 1.789 |  |
| ≥70 | 0 | – | 23 | 2.696 ± 2.363 |  | 3 | | 0.333 ± 0.577 | 31 | 2.581 ± 2.126 |  |
| Subtotal | 72 | 1.056 ± 1.221 | 106 | 2.462 ± 2.899 | <0.001 | 110 | | 1.036 ± 1.180 | 67 | 2.060 ± 1.938 | <0.001 |
| Total | 1.893 ± 2.462 | | | |  | 1.424 ± 1.587 | | | | |  |

^†^One study subject who had mean and maximum carotid intima media thickness of 2 mm was excluded as an outlier.

Table S4. Univariate predictors of subclinical atherosclerosis

|  | **Men** |  | **Women** |  |
| --- | --- | --- | --- | --- |
| **Factor** | **Univariate OR** | **p-value** | **Univariate OR** | **p-value** |
| Age (years) | 1.050 (1.017-1.084) | 0.003 | 1.061 (1.025-1.098) | 0.001 |
| Smoking (current or past)^†^ | 0.742 (0.312-1.764) | 0.500 | - | - |
| Duration of diabetes (year) | 1.030 (0.977-1.085) | 0.278 | 1.114 (1.043-1.190) | 0.001 |
| Family history of premature CHD  (yes vs no) | 1.016 (0.263-3.930) | 0.982 | 0.500 (0.129-1.942) | 0.317 |
| Waist circumference (cm) | 0.975 (0.929-1.023) | 0.299 | 1.038 (1.001-1.077) | 0.044 |
| SBP (mmHg) | 1.000 (0.975-1.026) | 0.984 | 1.011 (0.987-1.035) | 0.378 |
| DBP(mmHg) | 0.978 (0.944-1.013) | 0.220 | 0.980 (0.945-1.016) | 0.267 |
| hsCRP (mg/dL) | 1.029 (0.919-1.153) | 0.617 | 1.018 (0.865-1.199) | 0.828 |
| HDL-C (mg/dL) | 0.989 (0.960-1.019) | 0.482 | 0.997 (0.972-1.022) | 0.796 |
| TG (mg/dL) | 0.761 (0.997-1.002) | 0.761 | 0.999 (0.995-1.002) | 0.458 |
| LDL-C (mg/dL) | 1.004 (0.992-1.017) | 0.487 | 0.995 (0.984-1.007) | 0.401 |
| Total cholesterol (mg/dL) | 1.001 (0.990-1.011) | 0.877 | 0.994 (0.985-1.003) | 0.192 |
| ApoB (mg/dL) | 1.005 (0.989-1.022) | 0.537 | 0.989 (0.974-1.005) | 0.180 |
| ApoA1 (mg/dL) | 0.999 (0.987-1.011) | 0.888 | 1.001 (0.988-1.013) | 0.916 |
| Urine microalbumin (mg/day) | 0.999 (0.998-1.001) | 0.307 | 1.000 (0.999-1.000) | 0.579 |
| Menopause (yes vs no) | – |  | 2.779 (1.319-5.855) | 0.007 |
| Statin use (yes vs no) | 1.429 (0.715-2.855) | 0.312 | 1.346 (0.674-2.690) | 0.400 |
| ACE inhibition (yes vs no) | 0.927 (0.456-1.882) | 0.833 | 2.552 (1.242-5.245) | 0.011 |
| Antiplatelet use (yes vs no) | 1.846 (0.816-4.178) | 0.141 | 2.630 (1.085-6.377) | 0.032 |

^†^Frequency of past or current smokers in women was less than 5% and excluded in analysis among women.

ACE, angiostensin-converting enzyme; Apo, apolipoprotein; CHD, coronary heart disease; DBP, diastolic blood pressure; HDL, high density lipoprotein; hsCRP, high-sensitivity C-reactive protein; LDL, low density lipoprotein; OR, odds ratio; SBP, systolic blood pressure; TG, triglycerides

Table S5. Multivariate independent predictors of subclinical atherosclerosis

|  | **Men^†^** |  | **Women^‡^** |  |
| --- | --- | --- | --- | --- |
| **Factor** | **Multivariate OR** | **p-value** | **Multivariate OR** | **p-value** |
| Age (years) | 1.050 (1.017-1.084) | 0.003 | 1.042 (0.993-1.094) | 0.093 |
| Duration of diabetes (years) |  |  | 1.098 (1.020-1.181) | 0.013 |
| Waist circumference (cm) |  |  | 1.051 (1.008-1.096) | 0.020 |
| Menopause (yes vs no) |  |  | 1.375 (0.497-3.803) | 0.539 |

^†^Pseudo R^2^ Cox and Snell = 0.056

^‡^Pseudo R^2^ Cox and Snell = 0.138

OR, odds ratio.

Table S6. Mean cIMT according to age and the status of statin use

| **Mean IMT** | **Statin user** | | | **Statin non-user** | | | **p-value** |
| --- | --- | --- | --- | --- | --- | --- | --- |
| **Age (years)** | **n** | **Mean** | **SD** | **n** | **Mean** | **SD** |  |
| <40 | 10 | 0.513 | 0.089 | 10 | 0.527 | 0.082 | 0.677 |
| 40–49 | 40 | 0.530 | 0.093 | 19 | 0.539 | 0.088 | 0.758 |
| 50–59 | 82 | 0.582 | 0.093 | 32 | 0.596 | 0.120 | 0.505 |
| 60–69 | 69 | 0.620 | 0.115 | 36 | 0.597 | 0.110 | 0.338 |
| ≥70 | 36 | 0.690 | 0.124 | 21 | 0.775 | 0.389 | 0.228 |
| Total | 237 | 0.596 | 0.116 | 118 | 0.613 | 0.204 | 0.358 |

SD, standard deviation

Figure S1. Receiver operating characteristic curve analysis of conventional risk scores in predicting subclinical atherosclerosis (p = 0.189)


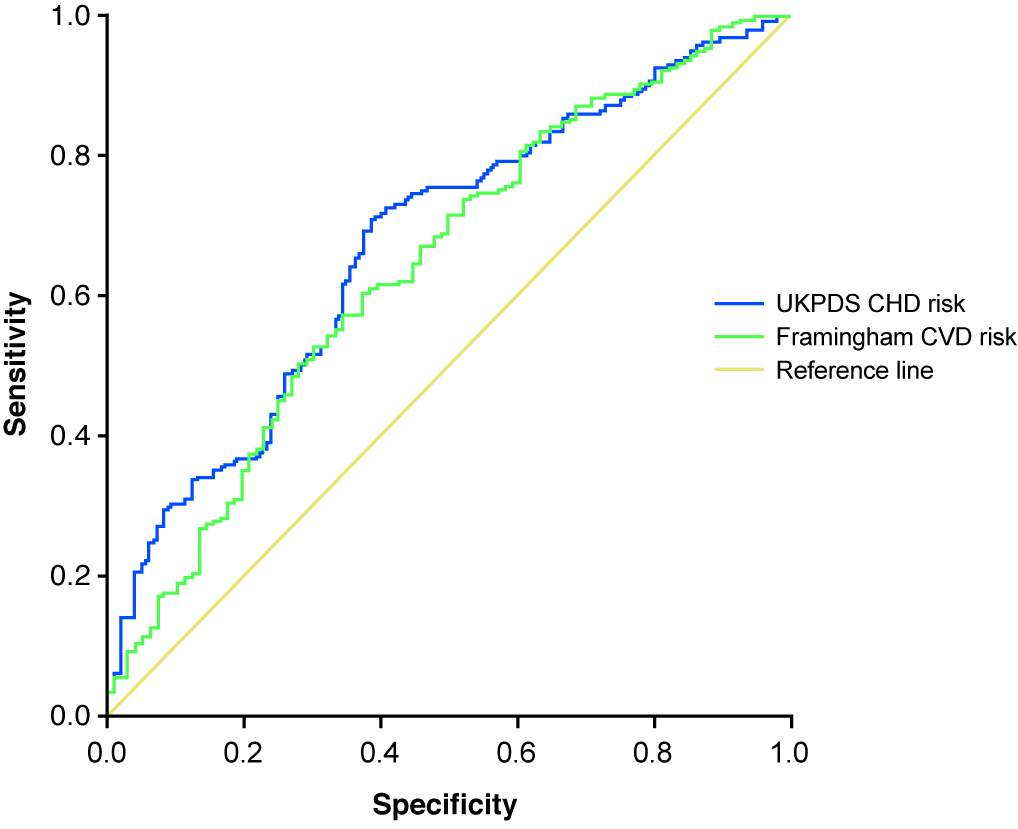


Diagonal segments are produced by ties.

CHD, coronary heart disease; CVD, cardiovascular disease; UKPDS, United Kingdom Prospective Diabetes Study
